# The International Prospective Glanzmann Thrombasthenia Registry: Pediatric Treatment and Outcomes

**DOI:** 10.1055/s-0039-1696657

**Published:** 2019-09-12

**Authors:** Rainer B. Zotz, Man-Chiu Poon, Giovanni Di Minno, Roseline D'Oiron, for the Glanzmann Thrombasthenia Registry Investigators

**Affiliations:** 1 Institute for Laboratory Medicine, Blood Coagulation and Transfusion Medicine (LBT), Düsseldorf, Germany; 2 Department of Hemostasis, Hemotherapy and Transfusion Medicine, Heinrich Heine University Medical Centre, Düsseldorf, Germany; 3 Departments of Medicine, Pediatrics and Oncology, University of Calgary, Calgary, Alberta, Canada; 4 Southern Alberta Rare Blood and Bleeding Disorders Comprehensive Care Program, Foothills Medical Centre, Calgary, Alberta, Canada; 5 Department of Clinical Medicine and Surgery, Regional Reference Center for Coagulation Disorders, Federico II University, Naples, Italy; 6 Center for Hemophilia and Rare Congenital Bleeding Disorders, University Hospitals Paris-Sud, AP-HP, Bicêtre Hospital, Le Kremlin-Bicêtre, France

**Keywords:** recombinant activated factor VII, Glanzmann thrombasthenia, autosomal dominant, pediatric, registry, observational study

## Abstract

**Background** Standard treatment for Glanzmann thrombasthenia (GT), a severe inherited bleeding disorder, is platelet transfusion. Recombinant activated factor VII (rFVIIa) is reported to be effective in GT with platelet antibodies and/or refractoriness to platelet transfusions.

**Methods** We evaluated rFVIIa effectiveness and safety for the treatment and prevention of surgical and nonsurgical bleeding in children <18 years old, with or without platelet antibodies and/or refractoriness, as reported in the GT Registry (GTR). Data were used from the GTR, an international, multicenter, observational, postmarketing study of rFVIIa that prospectively collected data on the treatment and outcomes of bleeds in patients with GT. Only patients with a diagnosis of congenital GT were included in the registry.

**Results** Between 2007 and 2011, 27 children were treated for 44 surgical procedures (minor: 36; major: 8); nonsurgical bleeds occurred in 104 patients (599 episodes: severe, 145; moderate, 454; spontaneous, 423; posttraumatic, 176). The effectiveness of treatment for minor procedures, major procedures, nonsurgical bleeds was 6/6, 1/1, and 75/84 for rFVIIa, 6/6, 2/2, and 64/76 for rFVIIa + antifibrinolytics (AF), 11/12, 1/1, and 162/214 for platelets ± AF, and 5/6, 0/3, and 33/45 for rFVIIa + platelets ± AF. In all, 25 adverse events were reported in children; no thromboembolic events were reported.

**Conclusion** For all patients, regardless of platelet antibody or refractoriness status, rFVIIa, administered with or without platelets (± AF), provided effective hemostasis with a low frequency of adverse events in surgical, as well as nonsurgical, bleeding in patients with GT.

clinicaltrials.gov identifier: NCT01476423.

## Introduction

Glanzmann thrombasthenia (GT) is an autosomal platelet function disorder caused by a quantitative or qualitative defect of the platelet membrane glycoprotein IIb–IIIa (integrin αIIbβ3) complex. 1 2 With a prevalence of 1:1 million, GT is rare, although the prevalence is higher in areas where marriage between relatives is common. It manifests clinically as an increased tendency to spontaneous bleeds (e.g., epistaxis, hematoma, menorrhagia, and bleeding complications) and also during and after surgery. 2 3

The standard treatment for GT is platelet transfusion (PT), which carries the risk of development of antibodies (AB) to αIIbβ3 and/or human leukocyte antigen (HLA). Possible alternatives to PT include the administration of antifibrinolytics (AF), local hemostatic agents, and bone marrow transplantation. In addition, recombinant activated factor VII (rFVIIa) can be used for the treatment and prevention of bleeding episodes, and for surgery, in patients with GT and AB, as well as past or present platelet refractoriness. Nevertheless, this off-label use of rFVIIa in patients with GT who have bleeds is increasing, mainly because of its convenience and to avoid alloimmunization to platelets. Data from an international survey of patients with GT who have bleeding episodes or hemorrhagic surgery complications supported a preliminary suggestion of an optimal rFVIIa dosing regimen for the treatment of moderate or severe bleeding episodes (≥80 μg/kg given at intervals of ≤2.5 hours for ≥3 doses). 4 Based on these survey results, rFVIIa was approved by the European Medicines Agency (EMA) in 2004 for use in patients with GT who have AB and past or present platelet refractoriness.

As the prevalence of GT is so low, data are not readily available for different subgroups of patients. Particularly rare are data on the treatment of GT in children, for both surgical and nonsurgical bleeding; though case studies are available, no large pediatric studies have been conducted. The GT Registry (GTR) was an international, multicenter, observational registry that focused on both adults and children. As such, it is one of the first efforts to provide pediatricians with comprehensive information on the effectiveness and safety of rFVIIa in children. This article summarizes new GTR data and aims to evaluate rFVIIa effectiveness and safety as a treatment for young patients with GT. Treatment results for all GTR patients have been published previously. 5 6

## Methods

The GTR was an international, multicenter, observational study on the effectiveness and safety of rFVIIa in patients with GT (ClinicalTrials.gov identifier: NCT01476423), prospectively collecting data on the treatment and outcomes of bleeds. Data were entered into the GTR from 2007 until its closure in 2011, using a customized, Web-based collecting tool. Treatment was based on local clinical practice rather than a set protocol, and performed in accordance with general data protection laws and local country requirements for conducting observational studies. 5 6 Centralized data management was overseen by Novo Nordisk and an external expert panel comprising four hemostasis physicians from Europe and North America.

### Patients

The analyses focused on patients <18 years old, and only a diagnosis of congenital GT was included in the GTR. Patients with acquired thrombasthenic states caused by autoimmune disorders or medications were excluded. Refractoriness and the presence of AB were coded initially and assessed periodically as deemed necessary by the investigator. Because tests for AB may not have been available at all centers, AB may also have been present in patients classified as having refractoriness only. For GT definitions, as well as key terms regarding bleeding, surgery, and definitions of effectiveness (effective, partially effective, ineffective), see Supplementary Table S1 .

### Ethics Committee Approvals

The GTR was conducted in accordance with the Declaration of Helsinki and Guidelines for Good Pharmacoepidemiology Practices. Each participating center complied with local regulations. Ethical and/or regulatory approval was obtained before data entry into the registry, as required. Signed informed consent to participate was obtained from all parents/legal guardians of the patients.

### Statistical Methods

The effectiveness analysis was based on all children in the GTR and treatment-allocated bleeds for which the efficacy endpoint was known. All children and bleeding episodes were included in the safety analysis. The effectiveness data were summarized using numerical variables (mean, standard deviation, median, maximum, and minimum), while categorical variables were summarized as numbers and percentages. No formal statistical comparisons were performed. No subdivision into age groups was performed because patient numbers would be too low to yield meaningful results.

## Results

### GTR Enrollment and Composition of Datasets

Details of recruitment into the GTR and the safety and effectiveness datasets for all surgical and nonsurgical bleeds have been reported previously for adults and children. 5 6 For children with GT, data were collected from 643 admissions (bleeding, 599; surgery, 44); 131 children with GT were enrolled from 45 sites in 15 countries from Africa, Asia, Europe, and North America. The safety analysis dataset included all 643 admissions, while effectiveness analyses were performed using data available from 590 admissions for nonsurgical bleeding and 44 surgical admissions.

### Clinical and Demographic Characteristics of the GTR Population Undergoing Surgery or with Nonsurgical Bleeds

The clinical and demographic characteristics of children who participated in the GTR and underwent surgery are provided in Table 1 . Of 44 invasive procedures reported in 27 patients, 36 (in 23 patients) were minor and 8 (in 8 patients) were major.

**Table 1:** Registry: clinical and demographic characteristics of the population <18 years old at admission

Variable	Nonsurgical patients ( *n* = 104)	Nonsurgical bleeding episodes ( *n* = 599)	Surgical patients ( *n* = 27)	Surgical episodes ( *n* = 44)
Males, *n* (%)	48 (46.2)	259 (43.2)	19 (70.4)	34 (77.3)
Females, *n* (%)	56 (53.9)	340 (56.8)	8 (29.6)	10 (22.7)
Age (y), mean ± SD	6.3 ± 4.8	6.6 ± 4.3	7.5 ± 4.5	7.9 ± 4.4
Males, mean ± SD	5.2 ± 4.4	5.1 ± 4.3	6.3 ± 4.0	7.2 ± 4.3
Females, mean ± SD	7.2 ± 5.0	7.7 ± 4.0	10.3 ± 4.8	10.0 ± 4.4
Age category, *n* (%)				
< 12 years	87 (83.7)	506 (84.5)	21 (77.8)	34 (77.3)
12–17 years	17 (16.4)	93 (15.5)	6 (22.2)	10 (22.7)
Body weight (kg), mean ± SD	21.2 ± 14.3	22.3 ± 13.3	30.3 ± 20.8	31.6 ± 20.8
Males, mean ± SD	19.3 ± 14.5	20.0 ± 14.1	26.3 ± 17.1	30.0 ± 19.8
Females, mean ± SD	22.8 ± 14.0	24.2 ± 12.4	39.9 ± 26.6	37.0 ± 24.2
Type of disease, *n* (%)				
Type 1	26 (25.0)	234 (39.1)	9 (33.3)	13 (29.6)
Type 2	12 (11.5)	34 (5.7)	1 (3.7)	1 (2.3)
Variant	3 (2.9)	8 (1.3)	–	–
Unknown type	63 (60.6)	323 (53.9)	17 (63.0)	30 (68.2)
History of antiplatelet AB and/or refractoriness to platelets				
AB (AB confirmed, refractoriness status unknown)	11 (10.6)	85 (14.2)	5 (18.5)	12 (27.3)
AB + refractoriness (both confirmed)	5 (4.8)	77 (12.9)	4 (14.8)	5 (11.4)
All AB a	16	162	9	17
Anti-αIIbβ3	13 (81.3)	64 (39.5)	7 (77.8)	13 (76.5)
Antihuman leukocyte antigen	2 (12.5)	9 (5.6)	1 (11.1)	3 (17.7)
Other	5 (31.3)	56 (34.6)	2 (22.2)	3 (17.7)
Refractoriness (refractoriness confirmed, AB not confirmed)	3 (2.9)	37 (6.2)	1 (3.7)	1 (2.3)
No confirmed history of antiplatelet AB and/or refractoriness to PT	85 (81.7)	400 (66.8)	17 (63.0)	26 (59.1)

Abbreviations: AB, antibodies; PT, platelet transfusions; SD, standard deviation.

a Number of types of AB may not add up to number of all AB, since type of AB was not always registered, or more than one type of AB was registered.

Of the 599 admissions for nonsurgical bleeds in children, 145 (24.2%) were classified as severe and 454 (75.8%) as moderate, while 423 bleeds (70.6%) were spontaneous and 176 (29.4%) were classified as being posttraumatic (see Supplementary Table S2 for a detailed description of the bleeding episodes reported). Clinical and demographic characteristics of the patients who experienced nonsurgical bleeds are presented in Table 1 .

### Surgical Bleeding—Minor Procedures

#### Treatment and Outcome

Of the 36 minor surgical procedures performed, dental procedures were most common (25/36; 69.4%), followed by nasal procedures (4/36; 11.1%). Most minor procedures ( Table 2A ) were treated with rFVIIa, either alone (6/36; 16.7%), with AF (6/36; 16.7%), or PTs (12/36; 33.3%). Data on the number of minor procedures rated as “effective” (see definition in Supplementary Table S1 ) for the different treatments (overall and stratified according to the status of platelet AB and platelet refractoriness) are provided in Table 2A .

**Table 2:** Children <18 years old: treatments rated “effective” for (A) surgical bleeds and (B) nonsurgical bleeds

**A: Surgical bleeds**						
**Surgical category/clinical status**	**rFVIIa** **number effective/total number (%)**	**rFVIIa + AF** **number effective/total number (%)**	**PT ± AF** **number effective/total number (%)**	**rFVIIa + PT ± AF** **number effective/total number (%)**	**AF** **number effective/total number (%)**	**Overall** **number effective/total number (%)**
Minor procedures ( *n* = 36)						
No AB/No refractoriness ( *n* = 18)	3/3 (100.0)	2/2 (100.0)	5/5 (100.0)	2/2 (100.0)	2/6 (33.3)	14/18 (77.8)
AB + refractoriness ( *n* = 5)	1/1 (100.0)	2/2 (100.0)	–	2/2 (100.0)	–	5/5 (100.0)
Refractoriness only ( *n* = 1)	–	–	–	0/1 (0.0)	–	0/1 (0.0)
AB only ( *n* = 12)	2/2 (100.0)	2/2 (100.0)	6/7 (85.7)	1/1 (100.0)	–	11/12 (91.7)
Total ( *n* = 36)	6/6 (100.0)	6/6 (100.0)	11/12 (91.7)	5/6 (83.3)	2/6 (33.3)	30/36 (83.3)
Major procedures ( *n* = 8)						
No AB/No refractoriness ( *n* = 8)	1/1 (100.0)	2/2 (100.0)	1/1 (100.0)	0/3 (–)	1/1 (100.0)	5/8 (62.5)
AB + refractoriness ( *n* = 0)	–	–	–	–	–	–
Refractoriness only ( *n* = 0)	–	–	–	–	–	–
AB only ( *n* = 0)	–	–	–	–	–	–
Total ( *n* = 8)	1/1 (100.0)	2/2 (100.0)	1/1 (100.0)	0/3 (–)	1/1 (100.0)	5/8 (62.5)
**B: Nonsurgical bleeds**						
**Variable**	**rFVIIa** **number effective/total number (%)**	**rFVIIa + AF** **number effective/total number (%)**	**PT ± AF** **number effective/total number (%)**	**rFVIIa + PT ± AF** **number effective/total number (%)**	**AF** **number effective/total number (%)**	**Overall** ** number effective/total number (%) a**
Bleed type						
Spontaneous bleeding ( *n* = 420)	29/34 (85.3)	43/54 (79.6)	129/173 (74.6)	28/38 (73.7)	102/121 (84.3)	331/420 (78.8)
Posttraumatic bleeding ( *n* = 170)	46/50 (92.0)	21/22 (95.4)	33/41 (80.5)	5/7 (71.4)	45/50 (90.0)	150/170 (88.2)
Bleed severity						
Severe bleeds ( *n* = 144)	–	16/23 (69.6)	50/62 (80.6)	11/21 (52.4)	33/38 (86.8)	110/144 (76.4)
Moderate bleeds ( *n* = 446)	75/84 (89.3)	48/53 (90.6)	112/152 (73.7)	22/24 (91.7)	114/133 (85.7)	371/446 (83.2)
Clinical status						
No AB/refractoriness ( *n* = 395)	66/73 (90.4)	34/37 (91.9)	100/121 (82.6)	22/26 (84.6)	118/138 (85.5)	340/395 (86.1)
AB + refractoriness ( *n* = 77)	3/4 (75.0)	4/5 (80.0)	30/57 (52.6)	5/6 (83.3)	5/5 (100.0)	47/77 (61.0)
Refractoriness only confirmed ( *n* = 37)	2/3 (66.7)	13/15 (86.7)	4/5 (80.0)	2/5 (40.0)	8/9 (88.9)	29/37 (78.4)
AB only confirmed ( *n* = 81)	4/4 (100.0)	13/19 (68.4)	28/31 (90.3)	4/8 (50.0)	16/19 (84.2)	65/81 (80.2)

Abbreviations: AB, antibodies; AF, antifibrinolytics (e.g., tranexamic acid); PT, platelet transfusions; rFVIIa, recombinant activated factor VII.

a Effectiveness outcome was missing for nine nonsurgical bleeds. Missing data are categorized as follows: spontaneous bleeding ( *n* = 3), posttraumatic bleeding ( *n* = 6); by treatment: rFVIIa ( *n* = 2), rFVIIa + AF ( *n* = 2), PT ± AF ( *n* = 1), rFVIIa + PT ± AF ( *n* = 1), AF ( *n* = 3); by severity: severe bleeds ( *n* = 1), moderate bleeds ( *n* = 8).

#### Treatment with rFVIIa

Data on rFVIIa use were available for 18 minor procedures treated with rFVIIa, rFVIIa + AF, or rFVIIa + PT ± AF; 100% of treatments were rated effective for rFVIIa (6/6) and rFVIIa + AF (6/6), and 83.3% (5/6) of treatments were rated effective for rFVIIa + PT ± AF ( Table 2A ). For all minor procedures, median rFVIIa dose was 175 μg/kg (range: 3.6–300; interquartile range [IQR]: 110 μg/kg), median number of doses was two (range: 1–20; IQR: 2) and median interval between doses was 2 hours (range: 2–6; IQR: 1 hour) ( Table 3 ).

**Table 3:** Children <18 years old: doses and duration of treatment with rFVIIa in the GTR stratified according to bleeding severity and to a history of AB/refractoriness for (A) surgical prophylaxis for bleeding ( *n* = 24) and (B) nonsurgical bleeds ( *n* = 210)

**A: Surgical prophylaxis for bleeding**										
**Surgical category/clinical status**	**Dose (μg/kg)**		**Number of doses**		**Cumulative dose (μg/kg)**		**Time interval between doses (h)**		**Duration of treatment (h)**	
	**Mean ± SD**	**Median** **IQR** **Range**	**Mean ± SD**	**Median** **IQR** **Range**	**Mean ± SD**	**Median** **IQR** **Range**	**Mean ± SD**	**Median** **IQR** **Range**	**Mean ± SD**	**Median** **IQR** **Range**
All minor procedures ( *n* = 18)	147.7 ± 55.5	175.0 110.0 3.6–300	4.2 ± 5.9	2.0 2.0 1–20	929.8 ± 2,166.5	185.0 120.0 3.6–8,544.0	2.6 ± 1.2	2.0 1.0 2.0–6.0	11.6 ± 16.5	5.0 3.0 2.0–54.0
All major procedures ( *n* = 6)	105.8 ± 27.9	90.0 30.0 79.0–180.0	10.7 ± 9.1	9.5 16.0 1.0–22.0	1,154.0 ± 1,019.9	1,035.0 1,530.0 180.0–2,608.0	5.5 ± 6.0	3.0 4.0 1.0–30.0	77.0 ± 51.3	72.5 82.0 24.0–139.0
No AB or refractoriness to PT ( *n* = 13)	118.3 ± 37.7	90.0 68.0 71.0–180.0	7.6 ± 8.1	3.0 13.0 1.0–22.0	1,300.8 ± 2,321.2	270.0 1,530.0 178.0–8,544.0	4.5 ± 5.1	3.0 2.0 1.0–30.0	38.2 ± 46.0	23.0 45.0 2.0–139.0
AB + refractoriness ( *n* = 11)	153.4 ± 64.6	200.0 110.0 3.6–300.0	3.6 ± 5.5	2.0 2.0 1.0–20.0	614.0 ± 1,332.5	270.0 175.0 3.6–4,617.0	2.7 ± 1.0	2.0 1.0 2.0–6.0	11.0 ± 19.0	5.0 4.0 2.0–54.0
**B: Nonsurgical bleeds**										
**Bleeding severity/clinical status**	**Dose (μg/kg)**		**Number of doses**		**Cumulative dose (μg/kg)**		**Time interval between doses (h)**		**Duration of treatment (h)**	
	**Mean ± SD**	**Median** **IQR** **Range**	**Mean ± SD**	**Median** **IQR** **Range**	**Mean ± SD**	**Median** **IQR** **Range**	**Mean ± SD**	**Median** **IQR** **Range**	**Mean ± SD**	**Median** **IQR** **Range**
Severe bleeding episodes ( *n* = 44)	91.4 ± 21.8	90.0 15.2 36.0–185.0	8.2 ± 11.3	5.5 6.0 1.0–59.0	1,499.4 ± 2,895.0	532.5 741.0 40.0–15,600.0	8.1 ± 14.1	3.0 4.0 1.0–114.0	77.6 ± 138.3	26.5 44.5 3.0–578.0
Moderate bleeding episodes ( *n* = 166)	96.9 ± 34.6	90.0 0.0 28.0–300.0	2.3 ± 1.9	2.0 2.0 1.0–10.0	220.2 ± 188.5	180.0 180.0 28.0–1,020.0	5.1 ± 8.6	3.0 1.0 1.0–70.0	17.7 ± 24.6	6.0 20.0 2.0–136.0
No AB or refractoriness to PT ( *n* = 138)	93.5 ± 34.5	90.0 0.0 36.0–290.0	2.1 ± 1.8	1.0 2.0 1.0–10.0	200.9 ± 178.6	128.2 180.0 40.0–1,020.0	5.3 ± 8.2	3.0 2.0 1.0–57.0	18.8 ± 25.0	5.0 23.0 2.0–136.0
AB + refractoriness ( *n* = 72)	94.8 ± 25.0	90.0 5.0 28.0–300.0	6.2 ± 9.3	3.0 5.0 1.0–59.0	1,049.0 ± 2,340.4	350.0 563.0 28.0–15,600.0	7.6 ± 13.6	3.0 3.0 1.0–114.0	58.0 ± 119.0	15.5 38.0 2.0–578.0

Abbreviations: AB, antibodies; GTR, Glanzmann Thrombasthenia Registry; IQR, interquartile range; PT, platelet transfusions; rFVIIa, recombinant activated factor VII; SD, standard deviation.

#### Treatment with Platelets

Treatment with PT ± AF was used in 12 minor procedures, with 91.7% of treatments overall being rated effective ( Table 2A ). This treatment was rated effective in all five procedures in the no AB/no refractoriness group and in six of seven procedures in the AB-only group. For the six minor procedures treated with rFVIIa + PT ± AF, 83.3% were rated effective overall ( Table 2A ).

#### Ineffective Treatments

One of the 36 minor procedures was rated “ineffective” (no AB/no refractoriness; AF only) using PT ± AF.

### Surgical Bleeding—Major Procedures

#### Treatment and Outcome

Of the eight major surgical procedures performed, gastrointestinal and circumcision procedures were most common ( *n* = 3; 37.5% each). Major procedures were treated most frequently with rFVIIa + PT ± AF ( *n* = 3; 37.5%). All major procedures occurred in the no AB/no refractoriness group. Effectiveness ratings for the different treatments for major procedures (overall and stratified according to the status of platelet AB and platelet refractoriness) are provided in Table 2A .

#### Treatment with rFVIIa

Data on rFVIIa use were available for analysis in six major procedures treated with rFVIIa, rFVIIa + AF, or rFVIIa + PT ± AF, with 100% of treatments rated effective for rFVIIa (1/1) and rFVIIa + AF (2/2); 0% (0/3) of treatments were rated effective for rFVIIa + PT ± AF ( Table 2A ). For all major procedures, the median rFVIIa dose was 90 μg/kg (range: 79–180); median dose interval was 3 hours (range: 1–30 hours). The median number of doses was 9.5 (range: 1–22) and the median cumulative rFVIIa dose was 1,035 μg/kg (range: 180–2,608) ( Table 3 ).

#### Treatment with Platelets

The effectiveness outcome for major procedures treated with platelets (PT ± AF and rFVIIa + PT ± AF) was available for four cases ( Table 2A ). Of these, the one treated with PT ± AF was effective.

#### Partially Effective/Ineffective Treatments

Of the eight major procedures, three were rated as either partially effective ( *n* = 2) or ineffective ( *n* = 1) in patients treated with rFVIIa + PT ± AF with no AB/refractoriness. No postsurgical bleeding was reported following rFVIIa and/or platelet treatment for major procedures (as defined in Supplementary Table S1 ).

#### Safety Data

For surgery, one nonserious adverse event (AE) was reported in the GTR, which was an incidence of pyrexia in a male child without platelet AB or refractoriness, who was treated with rFVIIa + PT + AF (major surgery). It was considered unlikely that this AE was related to rFVIIa, and the patient recovered completely. Supplementary Table S3 summarizes the AEs reported in treated patients.

### Nonsurgical Bleeding

#### Treatment Dosing and Scheduling

Regardless of bleeding severity, the PT ± AF combination was the most commonly used treatment ( Supplementary Fig. S1 ). Of the 590 bleeding episodes included in the effectiveness assessment, 214 (36.3%) were treated with PT ± AF, 171 (29.0%) with AF only, 84 (14.2%) with rFVIIa alone, 76 (12.9%) with rFVIIa + AF, and 45 (7.6%) with rFVIIa + PT ± AF. Concomitant AF treatment was documented in 76/160 (47.5%) bleeds treated with rFVIIa, in 60/214 (28.0%) treated with PT, and in 36/45 (80.0%) treated with rFVIIa + PT.

Investigators reported that, for the 195/590 bleeds that occurred in patients with a history of antiplatelet AB and platelet refractoriness, AB only, or refractoriness only, PT ± AF was used in 93/195 (47.7%; 29 severe and 64 moderate), rFVIIa + AF in 39/195 (20.0%, 20 severe and 19 moderate), AF alone in 33/195 (16.9%, 2 severe and 31 moderate), rFVIIa + PT ± AF in 19/195 (9.7%, 15 severe and 4 moderate), and rFVIIa alone in 11/195 (5.6%, all moderate) bleeds. The use of rFVIIa, alone (3/37; 8.1%) or together with AF (15/37; 40.5%), to treat bleeds was most frequent in patients with a history of refractoriness alone, while AF was mainly used to treat bleeds that occurred in patients with no history of AB and/or platelet refractoriness (138/395; 34.9%) ( Table 2B ).

The number of rFVIIa doses, cumulative dose, and overall duration of treatment were greater in patients with severe bleeds than in those with moderate bleeds. They were also greater in patients with a history of AB and/or refractoriness to platelets versus patients without such medical history ( Table 3 ).

#### Evaluation of Treatment Effectiveness

Overall, treatment was judged as effective (see Supplementary Table S1 for definition) in 89.3% (75/84) of bleeds treated with rFVIIa alone, 86.0% (147/171) with AF alone, 84.2% (64/76) with rFVIIa + AF, 75.7% (162/214) with PT ± AF, and 73.3% (33/45) with rFVIIa + PT ± AF ( Table 2B ). For 73 moderate bleeds treated with rFVIIa (either alone, with AF, or with PT) for which information on treatment duration was available, the median total duration of rFVIIa treatment was 6 hours. For 36 severe bleeds treated with rFVIIa (either alone, with AF, or with PT) for which information on treatment duration was available, the median total duration of rFVIIa treatment was 26.5 hours. In addition, the median total duration of treatment was 4 hours for 20 bleeds with AB, 11 hours for 13 bleeds with AB + refractoriness, 5 hours for 57 bleeds without AB or refractoriness, and 34 hours for 19 bleeds with refractoriness.

#### Ineffective Treatment

Ineffective treatment was documented in 13 cases (2.2% of the total number of patients), comprising three severe bleeds and 10 moderate bleeds. Of these, seven had a history of AB/refractoriness. Regarding the initial treatment employed, 8 of the 13 cases were receiving AF, 3 were receiving PT ± AF, and 2 were receiving rFVIIa + AF.

#### Rebleeding

For 557 bleeds where data on rebleeding were available, 36 rebleeds in 17 patients were registered, comprising 15/136 (11.0%) severe bleeds, 21/421 (5.0%) moderate bleeds, 15/380 (3.9%) bleeds in patients without AB or refractoriness, and 21/177 (11.9%) bleeds in patients with a history of AB and/or refractoriness to platelets. Rebleeding occurred in 20/204 (9.8%) bleeds treated with PT ± AF, 9/42 (21.4%) treated with rFVIIa + PT ± AF, 1/78 (1.3%) treated with rFVIIa alone, and 3/69 (4.3%) treated with rFVIIa + AF.

#### Safety Data

For the nonsurgical bleeding results presented in this article, 24 AEs were reported (including six serious AEs; Supplementary Table S3 ). For bleeds where patients received rFVIIa, eight AEs occurred, five of which were serious (subarachnoid bleeding, septicemia, respiratory insufficiency, cardiac decompensation, and rebleeding/hematoma due to a fall) and three nonserious (bacterial infection, fever, and headache). All were judged by the investigators as unlikely to be related to rFVIIa treatment. For the subarachnoid bleed, it was not possible to confirm the time relationship between this serious AE and rFVIIa treatment, i.e., whether rFVIIa was used to treat the subarachnoid bleed or not, and whether this is related to a lack of efficacy for rFVIIa or not. No thromboembolic events or unexpected laboratory values were reported for any of the treatments for nonsurgical bleeds.

## Discussion

The GTR data reported here have been taken from the largest observational study on patients with GT and include information on the management of invasive procedures; the shortcomings of the previous survey 4 have been addressed through consideration of the use of hemostatic agents other than rFVIIa. For the most part, this subgroup analysis confirmed previous insights. rFVIIa and other currently available treatments for bleeding in patients with GT were found to have good safety and effectiveness profiles in most children, in both surgical and nonsurgical bleeds. When compared with previous GTR analyses, overall there were no relevant differences between children and adults regarding effectiveness and safety of the studied treatment options (with the exception of PT for treatment of nonsurgical bleeds in children with AB and refractoriness, which was less effective than in adults). 5 6 The surgical patient subgroups included in the discussion below (e.g., patients with or without AB/refractoriness) were based on low patient numbers ( Table 2 ).

### Treatment with rFVIIa

In general, rFVIIa, alone or with AF, was used more frequently than platelets (PT ± AF) in surgical patients with AB and refractoriness, in surgical patients with major procedures, in nonsurgical patients with refractoriness only, and in nonsurgical patients with posttraumatic bleeding. The numbers receiving rFVIIa ± AF were similar in both surgical patients without AB/refractoriness and those with minor procedures overall. In all other subgroups, rFVIIa, alone or with AF, was used less often than platelets. The GTR results further indicate that rFVIIa has a good safety profile in patients with GT.

Assessment of rFVIIa dose and dosing schedule in this registry suggests that, in patients without AB or refractoriness, rFVIIa 90 to 120 μg/kg given at approximately 2 to 4-hour intervals (median: 2–3 hours) for ≥3 doses (median: 1–5.5, until effective hemostasis) could be used, with the first dose given immediately preoperatively in surgical patients. As reported by Poon et al, 6 this is similar to the regimen previously suggested for bleeding episodes (rFVIIa ≥80 μg/kg at intervals of ≤2.5 hours for ≥3 doses), 4 and is also similar to standard rFVIIa dosing in patients who have congenital hemophilia A or B with inhibitors. 7

A median rFVIIa dose of 90 μg/kg was used most frequently in nonsurgical patients with AB and/or refractoriness, and in surgical patients with neither of these (200 μg/kg was used in surgical patients with AB and/or refractoriness). The median dose interval was 3 hours, except in surgical patients with AB and/or refractoriness (for whom it was 2 hours). For severe bleeds, the number of doses reported was understandably higher than for moderate bleeds. In addition, severe bleeds were also associated with longer treatment duration and higher cumulative doses. Based on these results, rFVIIa at ≥90 μg/kg at intervals of ≤3 hours should be used, at least at the beginning of treatment. The subsequent number of doses required would need to be determined by the clinical situation and dosing should be continued until hemostasis is assured.

### Treatment with Platelets

PT was generally effective in covering surgical procedures and nonsurgical bleeds in patients, both with and without historic report of platelet AB and/or refractoriness. An exception is the much lower response rate for nonsurgical bleeds in children with AB and refractoriness (30/57, 52.6%) when compared with adults with AB and refractoriness (25/28, 89.3%). 5 Notably, some procedures in the AB-only group, as well as some nonsurgical bleeding episodes in patients with AB and/or refractoriness, had a successful outcome with PT ± AF ( Table 2 ). A possible explanation for this is that, in some patients with a history of AB, they were no longer present at the time of surgery and patients who had been refractory before were no longer. As discussed by Di Minno et al, 5 although comprehensive data on the natural history of inhibitors in GT are lacking, it is possible that the effectiveness of platelets in such a setting may be associated with the long time intervals between exposure to platelets in GT and possibly also to the transient nature of the inhibitors. Furthermore, Poon et al 6 suggested that the reason for this effectiveness may be that patients with HLA AB were receiving only HLA-matched platelets. Nevertheless, even if HLA-unmatched platelets are provided, prior data indicate that >50% of patients may not display refractoriness to PT. 8 9 10 As previously noted for the GTR in surgical intervention, 6 these observations suggest that treatment with platelets may be attempted when other agents are ineffective or not available—even in patients with a history of AB and/or refractoriness. 4 Due to the low numbers of surgical patients available, we cannot provide information on the effectiveness of treatment in refractory patients. Successful PT following removal of platelet AB by plasmapheresis 11 12 or immunoadsorption 13 has also been reported.

### Combined Treatment Using Platelets and rFVIIa

A key question is whether combined use of platelets and rFVIIa (rFVIIa + PT ± AF) has an advantage over rFVIIa (alone or with AF) or PT ± AF. 4 This pediatric analysis correlated with prior data reported from the GTR indicating that rFVIIa + PT ± AF was less effective than rFVIIa and rFVIIa + AF. 6 As previously suggested, based upon the observational nature of the registry, 6 rFVIIa + PT ± AF may have been used in patients with particularly difficult or challenging clinical situations and often when platelets may have been added to rFVIIa ± AF or rFVIIa added to PT ± AF. Further hemostatic agent(s) might have been added to the regimen when effectiveness was in doubt while using other hemostatic agents.

### Treatment with AF

The relative proportion of AF use for bleeds (both moderate and severe), as well as for surgical prophylaxis (both minor and major), was similar in both children and adults. 5 6 Nevertheless, in general, children with surgical procedures were administered AF more often than adults (7/44 [15.9%] vs. 5/159 [3.1%], respectively); rFVIIa was administered less often in children than adults (15/44 [34.1%] vs. 118/159 [74.2%], respectively). 6 For the treatment of bleeding episodes in particular, children with refractoriness or with AB were treated more often with AF than adults with the same status (refractoriness: 9/37 [24.3%] children vs. 0/16 [0%] adults; AB: 19/81 [23.5%] children vs. 11/52 [21.2%] adults). 5 AF were also used in children with spontaneous severe bleeding more often than in adults (36/131 [27.5%] vs. 9/67 [13.4%], respectively), although both subgroups were treated most often with PT (52/131 [39.7%] vs. 34/67 [50.7%], respectively). 5

The successful use of AF without platelets or rFVIIa was reported in 3/7 (42.9%) surgical procedures, and in 147/171 (86.0%) nonsurgical bleeding episodes. As noted previously for the GTR, 6 this may be explained by AF treatment commencing with the intention of using other systemic hemostatic agent(s), should hemostasis not be achieved. In addition, a less controlled data collection process versus clinical trials complicates interpretation or direct comparison of effectiveness between treatments. We do advise, however, that AF should not be recommended as the sole therapy during surgery in pediatric patients, particularly for major procedures, unless rFVIIa or platelet concentrates are available as back up. As noted by Di Minno et al, 5 nonsurgical bleeds may, on the other hand, be treated with AF first (possibly in the home setting), with the option of introducing other treatment (rFVIIa and/or platelets) during the bleeding episode if required.

### Safety

A potential concern with the use of rFVIIa in patients with GT is whether rFVIIa is thrombogenic. The pediatric data reported here suggest that all systemic hemostatic agents used had a good safety profile in surgical procedures for these patients. Among the 599 nonsurgical bleeding episodes in 104 patients and 44 procedures performed in 27 patients, no thromboembolic events were reported. Thus, rFVIIa appears to be a valid first-line treatment option for nonsurgical bleeds in pediatric patients with GT who are awaiting HLA-compatible platelet concentrates or for concentrates prepared from single-donor apheresis. Indeed, rFVIIa has been licensed for use in GT when platelets are not readily available. 14

### Limitations

As previously reported, 6 the major limitations of these GTR data are that they were not obtained using a defined treatment protocol in a randomized manner and treatment effectiveness and safety were not assessed at multiple, consistent, predefined time points. Furthermore, the frequent use of multiple agents in GT and delays in obtaining platelets make it particularly difficult to attribute effectiveness to any one or more products. Previously, Di Minno et al 5 noted the arbitrary classification of severe and moderate bleeds in nonsurgical bleeding as a further limitation. The use of these simple definitions, which are similar to the definitions of “major and minor bleeds” published in April 2005 by the Subcommittee on Control of Anticoagulation of the Scientific and Standardization Committee of the International Society on Thrombosis and Haemostasis, 15 was at the request of the EMA. Furthermore, as the coding of history of AB or refractoriness was performed at first admission and when the investigator considered appropriate, 6 the lack of documentation of specific antibody testing or refractoriness at the time of a particular episode limits the analysis (particularly for use of platelet-based regimens).

The rarity of GT also hinders the performance of formal clinical trials; pediatric data reported here represent the largest dataset available in the literature, including those in other databases. 16 17 These registry data represent real-life clinical practice and the standards of care at participating sites. Narrative information, as provided in the GTR, often gives additional useful insights that may differ from the coding of effectiveness at an earlier time point after surgery.

In summary, this post hoc analysis suggests that, when managing children with GT and surgical or nonsurgical bleeding episodes, rFVIIa, PT, and AF are valid first-line treatment options as they appear effective and safe. Given the selection bias in choice of treatment and adaption of treatment to clinical response, treatment strategies cannot be compared in observational studies. For first-line treatment of nonsurgical bleeds with AF, we suggest introducing other treatment options if AF proves insufficiently effective. For major surgical procedures, AF should only be used as a first-line therapy when rFVIIa or platelet concentrates are available as a backup treatment option. In cases of severe bleeding, a combined treatment with AF, rFVIIa, and platelet concentrates may be necessary. The available observations suggest that treatment with platelets may be attempted when other agents are ineffective or not available—even in patients with a history of AB and/or refractoriness. As there were no relevant differences between children and adults regarding effectiveness and safety of the studied treatment options (with the exception of PT in the treatment of nonsurgical bleeds in children with both AB and refractoriness), the treatment of children should not differ notably from that of adults. 5 6

## References

[1] Bellucci S Caen J Molecular basis of Glanzmann's thrombasthenia and current strategies in treatment Blood Rev 2002 16 03 193 202 12163005 10.1016/s0268-960x(02)00030-9

[2] George J N Caen J P Nurden A T Glanzmann's thrombasthenia: the spectrum of clinical disease Blood 1990 75 07 1383 1395 2180491

[3] Toogeh G Sharifian R Lak M Safaee R Artoni A Peyvandi F Presentation and pattern of symptoms in 382 patients with Glanzmann thrombasthenia in Iran Am J Hematol 2004 77 02 198 199 15389911 10.1002/ajh.20159

[4] Poon M C D'Oiron R Von Depka M Prophylactic and therapeutic recombinant factor VIIa administration to patients with Glanzmann's thrombasthenia: results of an international survey J Thromb Haemost 2004 2 07 1096 1103 15219192 10.1111/j.1538-7836.2004.00767.x

[5] Di Minno G Zotz R B d'Oiron R Bindslev N Di Minno M N Poon M C; Glanzmann Thrombasthenia Registry Investigators.The international, prospective Glanzmann Thrombasthenia Registry: treatment modalities and outcomes of non-surgical bleeding episodes in patients with Glanzmann thrombasthenia Haematologica 2015 100 08 1031 1037 26001793 10.3324/haematol.2014.121475 PMC5004418

[6] Poon M C d'Oiron R Zotz R B Bindslev N Di Minno M N Di Minno G; Glanzmann Thrombasthenia Registry Investigators.The international, prospective Glanzmann Thrombasthenia Registry: treatment and outcomes in surgical intervention Haematologica 2015 100 08 1038 1044 26001792 10.3324/haematol.2014.121384 PMC5004419

[7] Novo Nordisk. NovoSeven summary of product characteristics. Available at:http://www.ema.europa.eu/docs/en_GB/document_library/EPAR_-_Product_Information/human/000074/WC500030873.pdf. Accessed April 4, 2016

[8] Brand A Claas F H Voogt P J Wasser M N Eernisse J G Alloimmunization after leukocyte-depleted multiple random donor platelet transfusions Vox Sang 1988 54 03 160 166 3369136 10.1111/j.1423-0410.1988.tb03892.x

[9] Legler T J Fischer I Dittmann J Frequency and causes of refractoriness in multiply transfused patients Ann Hematol 1997 74 04 185 189 9174547 10.1007/s002770050280

[10] Trial to Reduce Alloimmunization to Platelets Study Group.Leukocyte reduction and ultraviolet B irradiation of platelets to prevent alloimmunization and refractoriness to platelet transfusions N Engl J Med 1997 337 26 1861 1869 9417523 10.1056/NEJM199712253372601

[11] Ito K Yoshida H Hatoyama H Antibody removal therapy used successfully at delivery of a pregnant patient with Glanzmann's thrombasthenia and multiple anti-platelet antibodies Vox Sang 1991 61 01 40 46 1949709 10.1111/j.1423-0410.1991.tb00925.x

[12] Vivier M Treisser A Naett M Glanzmann's thrombasthenia and pregnancy. Contribution of plasma exchange before scheduled cesarean section [in French] J Gynecol Obstet Biol Reprod (Paris) 1989 18 04 507 513 2778287

[13] Martin I Kriaa F Proulle V Protein A Sepharose immunoadsorption can restore the efficacy of platelet concentrates in patients with Glanzmann's thrombasthenia and anti-glycoprotein IIb-IIIa antibodies Br J Haematol 2002 119 04 991 997 12472579 10.1046/j.1365-2141.2002.03936.x

[14] NiaStase RT ^®^ . Product Monograph. Available at: http://www.novonordisk.ca/content/dam/Canada/AFFILIATE/www-novonordisk-ca/OurProducts/PDF/niastase-product-monograph.pdf. Accessed November 8, 2017

[15] Schulman S Kearon C; Subcommittee on Control of Anticoagulation of the Scientific and Standardization Committee of the International Society on Thrombosis and Haemostasis.Definition of major bleeding in clinical investigations of antihemostatic medicinal products in non-surgical patients J Thromb Haemost 2005 3 04 692 694 15842354 10.1111/j.1538-7836.2005.01204.x

[16] Chitlur M Ewing N Kraut E H Recombinant factor VIIa (rFVIIa) use in Glanzmann's thrombasthenia (GT) and other platelet disorders (OPDS): Hemophilia and Thrombosis Research Society (HTRS) registry data. [abstract] J Thromb Haemost 2011 9(Suppl. 2):340

[17] Lak M Scharling B Blemings A Evaluation of rFVIIa (Novo-Seven) in Glanzmann patients with thromboelastogram Haemophilia 2008 14 01 103 110 18070065 10.1111/j.1365-2516.2007.01592.x

