# Supplementary material for: The International Prospective Glanzmann Thrombasthenia Registry: Pediatric Treatment and Outcomes
Source: TH Open. 2019 Sep 12;3(3):e286–94. doi: 10.1055/s-0039-1696657 (PMC6742499; doi:10.1055/s-0039-1696657)
Supplement: Supplementary file 1 — Supplementary Material [file 10-1055-s-0039-1696657-s190008.pdf]

Supplementary Table S1 Definitions used in the GTR

General definitions	
Patients with GT	▪ Males and females, any age, with a life-long bleeding tendency and a prolonged bleeding time or a prolonged PFA closure time; impaired or absent platelet aggregation (e.g., ADP, epinephrine, collagen, arachidonic acid, thrombin [but normal ristocetin]); normal platelet counts and platelet morphology. Optional diagnostic criteria: impaired clot retraction; quantitative/qualitative evaluation of GP IIb/IIIa receptors (flow cytometry); identification of gene defects
Rebleeding	▪ A bleed starting ≥6 hours and <48 hours after the initial bleeding stopped
New bleeding episode	▪ Bleeding after an effective outcome of the initial prophylactic treatment, occurring ≤7 days after the last hemostatic treatment
Refractoriness to platelets (past or present)	▪ Bleeding during surgery despite an adequate amount[a] of platelet infusion and/or<br>▪ Persistence of bleeding despite an adequate amount[a] of platelet infusion and/or<br>▪ Rebleeding within 24 hours, despite an adequate amount[a] of platelet infusion
**Definitions specific to surgical bleeding**	
Major surgery	▪ A body cavity was entered<br>▪ A mesenchymal barrier (e.g., pleura, peritoneum, or dura) was crossed<br>▪ A fascial plane was opened<br>▪ An organ was removed<br>▪ Normal anatomy was operatively altered
Minor surgery	▪ Any invasive operative procedure in which only skin, mucous membranes, or superficial connective tissue was manipulated
Postsurgical bleeding	▪ Bleeding after an effective outcome for the initial prophylactic treatment for the surgical procedure, occurring ≤7 days after the last hemostatic treatment[b]
Effectiveness evaluations (surgery)	▪ *Effective*: normal hemostasis<br>▪ *Partially effective*: mild bleeding tendency<br>▪ *Ineffective*: excessive bleeding tendency
**Definitions specific to nonsurgical bleeding**	
Severe bleeding[c]	▪ Intracranial and/or resulting from severe trauma and/or symptomatic bleeding in a critical area, or compressing a vital organ or leading to blood transfusion (need for transfusion of ≥2 U of packed red cells)
Moderate bleeding[c]	▪ Bleeds requiring a systematic hemostatic treatment other than AF drugs, but not meeting the criteria for the definition of severe bleeds
Effectiveness evaluations (nonsurgery)	▪ *Effective*: bleeding stopped and hemostasis achieved for ≥6 hours<br>▪ *Partially effective*: bleeding decreased substantially but continued<br>▪ *Ineffective*: bleeding unchanged or worsened following treatment

Abbreviations: ADP, adenosine diphosphate; AF, antifibrinolytic; GP, glycoprotein; GT, Glanzmann thrombasthenia; GTR, Glanzmann Thrombasthenia Registry; PFA, platelet function assay.
Source: Adapted from Table 1 in Poon et al (2015)[6] and Supplementary Table 1 in Di Minno et al (2015).[5]
[a]An adequate amount is determined by the treating clinician.
[b]Bleeding occurring beyond 7 days after the last hemostatic treatment was considered to be a new bleeding episode unrelated to the surgical procedure or its prophylactic regimen.
[c]These post hoc definitions of "severe and moderate bleeds" (employed in response to requests from the EMA) are similar to the definitions of "major and minor bleeds" published in April 2005 by the Subcommittee on Control of Anticoagulation of the Scientific and Standardization Committee of the International Society on Thrombosis and Haemostasis.[15]

Supplementary Table S2 GTR: Nonsurgical bleeding treated episodes stratified according to post hoc severity categorization

	Moderate bleeding ($n = 454$)	Severe bleeding ($n = 145$)
Epistaxis	206 (45.4%)	73 (50.3%)
Menorrhagia	20 (4.4%)	19 (13.1%)
Oral	45 (9.9%)	12 (8.3%)
Easy bruising	165 (36.3%)	54 (37.2%)
Gum bleed	127 (28.0%)	43 (29.7%)
Subcutaneous hematoma	77 (17.0%)	13 (9.0%)
Muscle hematoma	17 (3.7%)	0 (0%)
Hematuria	8 (1.8%)	1 (0.7%)
Central nervous system	0 (0%)	1 (0.7%)
Hemarthrosis	10 (2.2%)	0 (0%)
Gastrointestinal	9 (2.0%)	17 (11.7%)
Hemorrhoidal	5 (1.1%)	3 (2.1%)
Hemoperitoneal	0 (0.0%)	1 (0.7%)
Other	53 (11.7%)	12 (8.3%)
Unknown	4 (0.9%)	2 (1.4%)

Abbreviation: GTR, Glanzmann Thrombasthenia Registry.

Supplementary Table S3 Children <18 years old: summary of AEs reported in treated patients in the GTR[a]

	Treatment including rFVIIa		Hemostatic treatment not including rFVIIa		Total	
	Patients, *n*	AEs	Patients, *n*	AEs	Patients, *n*	AEs
Surgical bleeding						
All, *n*	1	1	0	0	1	1
Serious AEs, *n*	0	0	0	0	0	0
Nonserious AEs, *n*	1	1	0	0	1	1
Nonsurgical bleeding						
All, *n*	4	8	7	16	9	24
Serious AEs, *n*	2	5	1	1	2	6
Nonserious AEs, *n*	3	3	7	15	9	18
Total[b]						
All, *n*	5	9	7	16	10	25
Serious AEs, *n*	2	5	1	1	2	6
Nonserious AEs, *n*	4	4	7	15	10	19

Abbreviations: AE, adverse event; GTR, Glanzmann Thrombasthenia Registry; rFVIIa, recombinant activated factor VII.
[a]A total of 32 AEs in 11 patients were reported in the GTR (children); 7 AEs were reported in 2 patients and were not associated with any bleeding episode or hemostatic treatment.
[b]As patients may have had both surgical and nonsurgical bleeding, and/or serious and nonserious AEs, some patients have been included in the patient counts more than once.

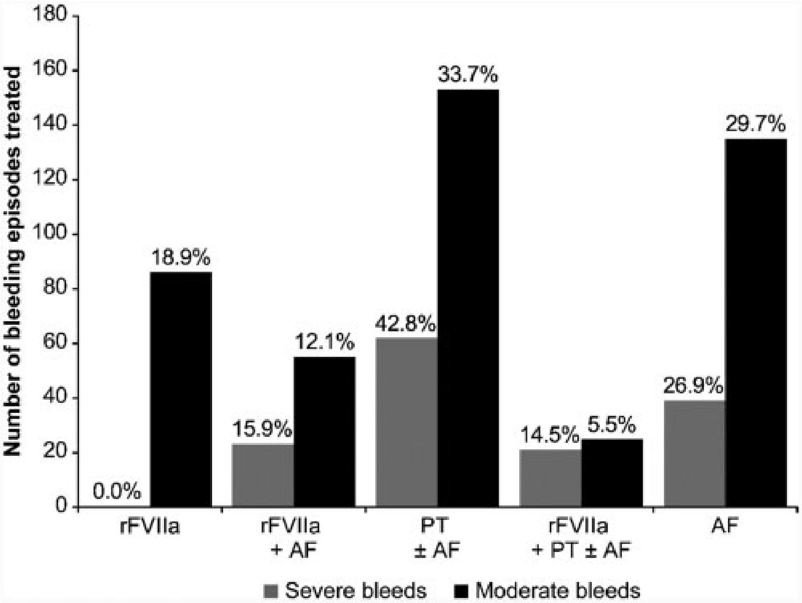

Supplementary Fig. S1 Treatment modalities in GTR stratified according to the severity of bleeding episode. The values provided above the bars represent the percentages of severe or moderate bleeds treated by each treatment. AF, antifibrinolytics; GTR, Glanzmann Thrombasthenia Registry; PT, platelet transfusions; rFVIIa, recombinant activated factor VII.
